# Case Report: Lasting complete response to pembrolizumab in mismatch repair-deficient cardiac sarcoma: a genomic characterization

**DOI:** 10.3389/fonc.2025.1485386

**Published:** 2025-04-03

**Authors:** Daniela A. Ferraro, Bettina Bisig, David C. Rotzinger, Fresia Pareja, Edoardo Missiaglia, Ioannis Voutsadakis, Krisztian Homicsko, Antonia Digklia

**Affiliations:** ^1^ Department of Medical Oncology, CHUV University Hospital, Lausanne, Switzerland; ^2^ Institute of Pathology, Department of Laboratory Medicine and Pathology, CHUV University Hospital, Lausanne, Switzerland; ^3^ Faculty of Biology and Medicine, University of Lausanne, Lausanne, Switzerland; ^4^ Department of Radiology, CHUV University Hospital, Lausanne, Switzerland; ^5^ Department of Pathology, Memorial Sloan Kettering Cancer Center, New York, NY, United States; ^6^ Algoma District Cancer Program, Sault Area Hospital, Sault Ste. Marie, ON, Canada; ^7^ Division of Clinical Sciences, Section of Internal Medicine, Northern Ontario School of Medicine, Sudbury, ON, Canada

**Keywords:** cardiac sarcoma, immune-checkpoint inhibitors (ICI), complete response (CR), predictive markers, mismatch repair deficiency (MMRd), microsatellite instability (MSI)

## Abstract

Sarcomas are traditionally considered “cold” tumors with poor response to immunotherapy. However, evidence accumulating over the last years shows that immune checkpoint inhibitors (ICIs) may have a role in selected sarcoma patients according to predictive markers. Here, we report the case of a woman diagnosed with a primary cardiac undifferentiated sarcoma. Following failure of standard first line chemotherapy, high-throughput sequencing (HTS) revealed a high tumor mutational burden (TMB), pathogenic mutations in *FAT1* and *NOTCH2* and a microsatellite instability (MSI)-associated signature. Immunohistochemistry confirmed mismatch repair-deficiency (MMRd) and abundant CD8+ tumor-infiltrating lymphocytes (TILs), in the absence of tertiary lymphoid structures. The patient was, therefore, treated with the ICI pembrolizumab, reaching a complete response that continues to persist at last follow-up, more than seven years from initial diagnosis and nearly six years from initiation of ICI treatment. This case illustrates the importance of performing HTS in rare sarcomas given the availability of efficient therapies, such as those for tumors displaying high TMB or MMRd/MSI. In agreement with other reports, it supports the contention that MMRd/MSI status and high numbers of TILs are valuable predictive markers of response to immunotherapy in sarcomas.

## Introduction

Immune check-point inhibitors (ICIs) have shown remarkable results in the treatment of various cancer types, but their efficacy in soft tissue sarcomas (STS) is low except for some subtypes, including undifferentiated pleomorphic sarcoma (UPS), alveolar soft part sarcoma, clear cell sarcoma, dedifferentiated liposarcoma (DDLPS) and angiosarcoma (1, 2). Similarly, in a recently published series of primary cardiac sarcomas the response to ICI was overall poor, although it was particularly dismal for patients with angiosarcoma, the most frequent primary cardiac sarcoma subtype (3, 4).

Tumor-infiltrating lymphocytes (TILs), tumor mutational burden (TMB), and PD-L1 expression are the most important predictive markers of response to ICI therapy in epithelial tumors (5, 6). Compared to highly immunogenic tumors such as melanoma or lung cancer, sarcomas usually display a low TMB (average of 2 mutations/Mb), low extent of TILs and low PD-L1 expression (7, 8).

Another more recently described predictive marker for ICIs is the presence of tertiary lymphoid structures (TLS) within the tumor or in its immediate proximity. TLS are lymphoid aggregates with features similar to B-cell follicles in lymph nodes, believed to have a relevant role in eliciting an antitumor immune response. Patients with abundant TLS have shown an increased survival and better response to ICIs in different cancer types, including STS. However, their prevalence in STS is low, in the range of 10% (9, 10). The genetic landscape of sarcomas is markedly heterogeneous, and rare sarcoma cases harbor a high TMB and/or high numbers of TILs. This is more common in UPS and leiomyosarcoma (LMS) and correlates with response to ICIs. Indeed, tumors exhibiting a high TMB are considered more immunogenic and more likely to respond to ICIs due to a greater abundance of tumor neo-antigens able to activate the immune system (11). High TMB may result from mismatch repair deficiency (MMRd), translating into a characteristic mutational signature consisting of high levels of microsatellite instability (MSI) across the genome. MSI signature, however, is rare in sarcomas, and has been reported in only 2% of patients (12, 13).

Pembrolizumab was the first PD-1 inhibitor approved by the American Food and Drug Administration (FDA) for MMRd/MSI tumors, initially in colon cancer and later in a tissue-agnostic manner (14–17).

Here, we present the case of a 73-year-old woman with an MMRd/MSI primary cardiac undifferentiated sarcoma who achieved a complete and durable response with the anti-PD1 inhibitor pembrolizumab after failure of standard first-line treatment.

## Case description

A 73-year-old woman presented in September 2016 with progressive dyspnea of several months of duration. Magnetic resonance imaging (MRI) revealed a left atrial tumor mass. A surgical resection of the lesion was performed one month later necessitating pulmonary valve and artery replacement. Histopathologic examination of the specimen led to the diagnosis of primary cardiac undifferentiated sarcoma, after exclusion of an intimal sarcoma in the absence of *MDM2* gene amplification (18). Post-operatively, the patient received no adjuvant therapy and was placed on radiologic surveillance. A cardiac MRI nine months later showed disease relapse with the presence of four solid intra-cardiac lesions in the inferior vena cava, in the right and left atria, and in the superior vena cava, without vascular compression and with a preserved cardiac function (left ventricular ejection fraction (LVEF) 51%). Positron emission tomography/computed tomography (PET/CT) confirmed the presence of hypermetabolic cardiac masses without evidence of metastasis. Brain MRI did not show metastatic brain disease.

**Figure 1:**
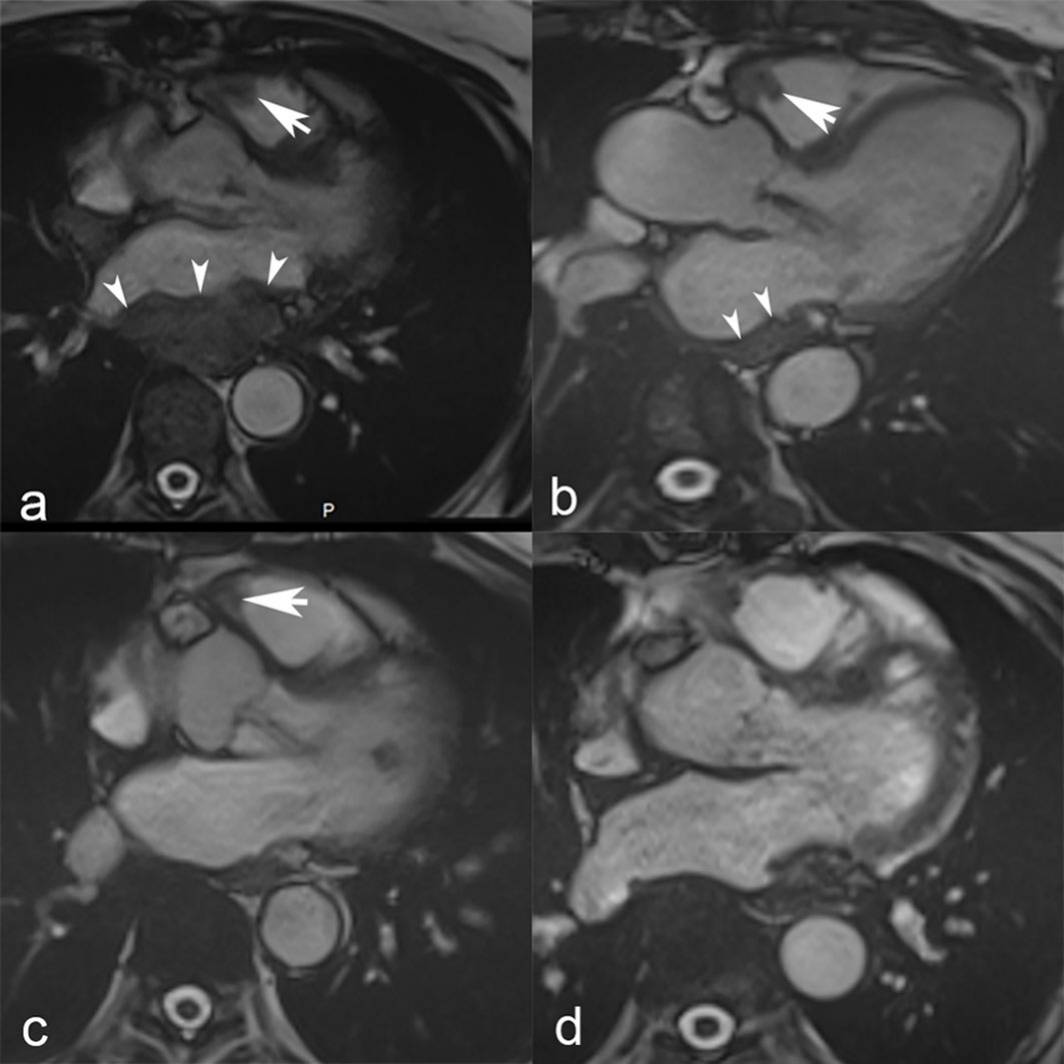
Cardiac MRI. Steady-state free precession cardiac MRI scans show masses in the right ventricular outflow tract (white arrow) and left atrium (white arrowheads) at baseline in March 2018 **(a)**. Nine months later, in September 2018 **(b)**, a follow-up MRI showed a slight decrease of the mass located in the right ventricular outflow tract (white arrow) and notable shrinkage of the left atrial mass that is merely visible as an atrial wall thickening (white arrowheads). Further tumor shrinkage of the right ventricular outflow tract mass (white arrow) was observed in May 2019 **(c)**, and ultimately complete response with no residual tumor in October 2020 **(d)**.

A first line therapy consisting of doxorubicine (75mg/m2 every 3 weeks) together with olaratumab (15 mg/kg, at day 1 and 8 every 21 days) (19) was administered starting in August 2017. Given an anaphylactic shock following the first dose, no antineoplastic agents were administered for 8 months. Progressive disease developed, and a cardiac MRI showed a partial occlusion of the inferior vena cava, masses in the right ventricular outflow tract, and atria (**Figure 1**).

At that time, in search for alternative treatment options, the initial tumor resection specimen was subjected to further predictive biomarker analyses.

Targeted high-throughput sequencing (HTS), covering the coding region of 394 cancer-related genes (1.24 megabases (Mb)), was performed in parallel on DNA extracted from the tumor tissue and matched constitutional DNA, using the MiSeq platform (Illumina). This analysis identified 17 somatic mutations (10 missenses, 6 frameshifts and 1 splice site mutation) (**Table 1**), providing an estimated TMB of 12.9 non-synonymous somatic mutations per Mb (splice site mutation excluded). Although this latter was not very high, the presence of a large proportion of transitions and small indels in homopolymeric sequences was suggestive of an MSI-associated mutational signature (23). Interestingly, a likely pathogenic somatic splice variant was detected at the intron 1/exon 2 boundary of *MLH1* (c.117-3C>G), and was associated with a copy gain of the mutated allele and a loss of heterozygosity (loss of the wild-type allele).

**Table 1:** Somatic mutations detected by high-throughput sequencing (394-gene panel).

Gene	Exon	Reference transcript	Mutations	Variant allele frequency	Coverage	Pathogenicity class^§^
*TP53*	7	NM_000546.5	c.742C>T, p.(Arg248Trp)	46%	241X	5
*MLH1*	2	NM_000249.3	c.117-3C>G (splice site)	69%	464X	4
*FAT1*	10	NM_005245.3	c.8799del, p.(Gly2934Valfs*3)	33%	878X	4
*NOTCH2*	34	NM_024408.3	c.6650_6651del, p.(Val2217Alafs*26)	17%	846X	4
*BAP1*	6	NM_004656.3	c.436A>T, p.(Arg146Trp)	53%	481X	3
*CBFB*	1	NM_022845.2	c.54del, p.(Phe18Leufs*4)	29%	519X	3
*PTPRT*	3	NM_133170.3	c.444G>T, p.(Lys148Asn)	21%	840X	3
*PNRC1*	1	NM_006813.2	c.332C>A, p.(Pro111His)	20%	2290X	3
*LRP6*	6	NM_002336.2	c.1022G>A, p.(Arg341His)	19%	937X	3
*KAT6A*	17	NM_006766.4	c.5303C>T, p.(Ala1768Val)	17%	809X	3
*NCOR2*	25	NM_006312.5	c.3178del, p.(Arg1060Valfs*3)	14%	380X	3
*PRKDC*	69	NM_006904.6	c.9554G>A, p.(Arg3185Gln)	14%	409X	3
*CDK12*	2	NM_016507.3	c.1139G>A, p.(Arg380His)	14%	639X	3
*KMT2D*	11	NM_003482.3	c.3161C>T, p.(Pro1054Leu)	11%	849X	3
*RSPO2*	3	NM_178565.4	c.205C>T, p.(Arg69Cys)	11%	1297X	3
*ZFHX3*	10	NM_006885.3	c.9589del, p.(Gln3197Serfs*44)	5%	552X	3
*GLI1*	8	NM_005269.2	c.821del, p.(Gly274Alafs*6)	5%	2521X	3

^§^Pathogenicity classes according to ACGS (Association for Clinical Genetic Science): class 5, pathogenic; class 4, likely pathogenic; class 3, of uncertain significance.

Estimated tumor cell content: 50%.

Mutation nomenclature: according to HGVS (Human Genome Variation Society).

**Figure 2:**
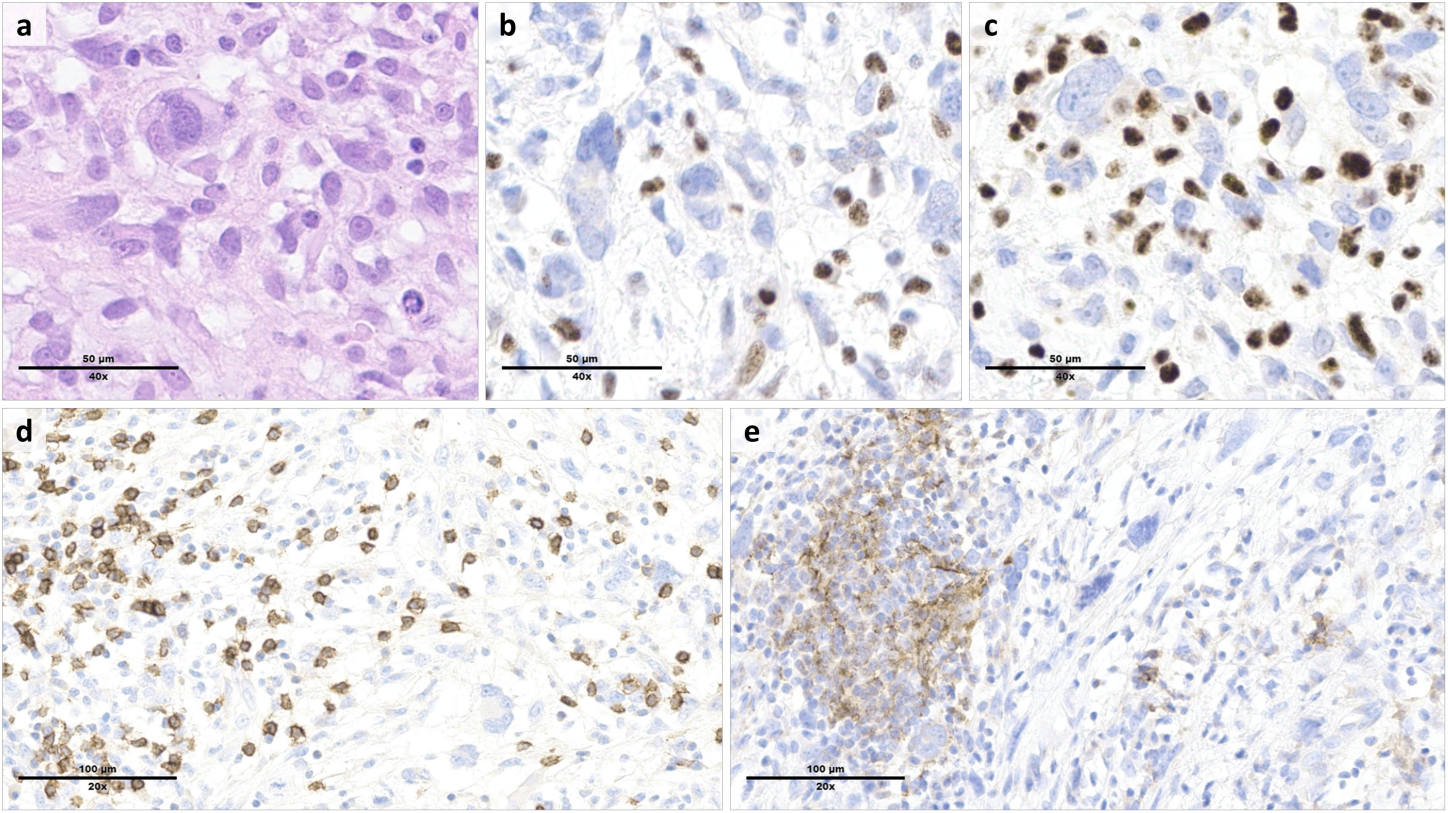
Morphologic and immunophenotypic features of the tumor resection specimen. Histologic examination of the cardiac mass showed a high-grade proliferation of tumor cells ranging from spindle-shaped to pleomorphic (**(a)**, hematoxylin & eosin). By immunohistochemistry, the tumor cells were characterized by a loss of MLH1 **(b)** and PMS2 **(c)** expression, which was preserved in the reactive background. Tumor infiltrating lymphocytes included numerous CD8+ T cells **(d)**. PD-L1 staining was negative in the tumor cells, but positive in a subset of tumor-associated immune cells **(e)**.

Immunostains for MMR proteins confirmed a loss of nuclear expression of MLH1 and PMS2 in the tumor cells (**Figures 2a–c**), with retained MSH2 and MSH6. The *MLH1* gene promoter, evaluated by pyrosequencing after bisulfite conversion, did not show hypermethylation. No tertiary lymphoid structures (TLS) were detected based on standard hematoxylin & eosin-stained slides. TILs were abundant, with a mean of >20 intratumor CD8+ T cells per high power field (400x magnification) (**Figure 2d**). The tumor cells were negative for PD-L1 (tumor proportion score (TPS) <1%), while a subset of tumor-associated immune cells were positive (combined positive score (CPS) >20) (**Figure 2e**).

Other relevant findings of HTS included a pathogenic missense mutation in exon 7 of *TP53*, and likely pathogenic frameshift deletions in exon 10 of *FAT1* and exon 34 of *NOTCH2*.

Based on these results, the patient started treatment with the anti-PD-1 antibody pembrolizumab (200 mg every 3 weeks), in May 2018. Pembrolizumab was administrated for 14 cycles, when it was discontinued due to autoimmune colitis grade 3 and adrenal insufficiency grade 3 secondary to hypophysitis, which needed the introduction of steroids. Under steroid therapy the immune adverse events resolved after a few weeks. Given the grade 3 of the immune-adverse events, the treatment with pembrolizumab was definitively stopped. The adrenal insufficiency was substituted with hydrocortisone.

**Figure 3:**
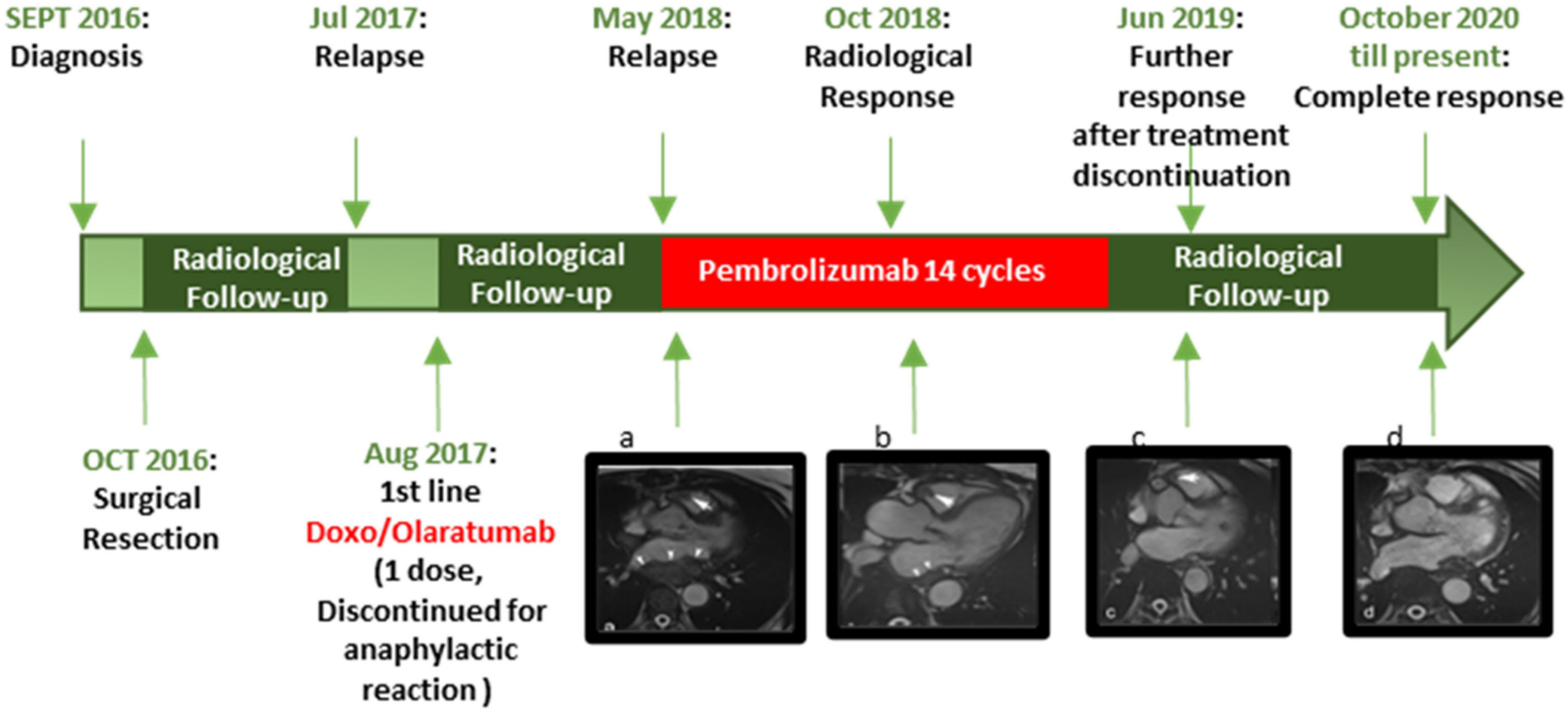
Timeline of principal episodes of the case described.

The radiological follow-up during treatment showed a partial response (PR) according to RECIST criteria 6 months after the initiation of pembrolizumab. Following discontinuation of pembrolizumab, the radiological follow-up was performed with cardiac MRI every three months. Complete response (CR) was achieved several months after discontinuation in October 2020, and no progressive disease was observed until the latest follow-up (March 2024) (**Figure 3**).

## Discussion

STS are a heterogeneous group of over 100 different entities with distinct clinical and biological behaviors also due to the very complex genetic landscape of these diseases (20).

Currently, the main approach guiding decisions on sarcoma treatments continues to be histology-based, and no predictive biomarkers are taken into consideration, mainly due to the vast histologic and biologic heterogeneity and the lack of targetable oncogenic drivers in the most common subtypes. Chemotherapy remains the main systemic treatment with, however, a low response rate (around 12% to 25%). Low response rates result in an overall survival of patients with metastatic disease being limited to a median of about 18 months (21, 22). This dismal prognosis calls for a change of approach aiming not only to discover new therapeutic agents, but also to validate predictive biomarkers for a more rational and effective therapeutic choice.

In the past 10 years, immunotherapy revolutionized cancer treatment both by improving the outcomes in several types of advanced cancer, and by imposing a new way of thinking in oncology (24). In addition, the new approach underlined the need to identify new biomarkers to efficiently select patients for these treatments.

Traditionally, sarcomas are considered non-immunogenic tumors (1), although, due to their extensive molecular heterogeneity between different subtypes, some variability is observed. The SARC-028 trial has shown the effectiveness of ICI therapy in a subset of sarcoma patients, and identified histologies of STSs with the highest responses. These included UPS with an overall response rate (ORR) of 40% (4 of 10 patients), and DDLPS with an ORR of 20% (2 of 10). Notably, one patient with UPS reached a complete response (1, 2).

The discovery and validation of predictive biomarkers are crucial for personalizing therapy, and the field of immunotherapy is no exception. Traditionally, the biomarker field for immunotherapies has focused on single cancer intrinsic factors or immune-specific markers, although given the complexity of the interaction between tumor and stroma, a combinatorial approach could be more advantageous. Predictive biomarkers for ICI immunotherapy proposed in clinical practice include PD-L1 expression, TILs, TMB and MMRd/MSI status and more recently TLS (24, 25).

For the patient we report here, who had a severe reaction to chemotherapy and refused further treatment, the choice of using ICI therapy was biomarker-driven. Extensive analysis of the cardiac tumor specimen displayed several markers known to favor responses to ICIs, despite the absence of TLS. The tumor harbored a high TMB (> 10 mutations/Mb) and a MMRd/MSI profile. Moreover, the tumor displayed an extensive infiltrate by CD8+ T cells, and PD-L1, although negative in the tumor cells, was expressed in the tumor immune microenvironment. Based on these biomarkers and given the paucity of other treatment options acceptable to the patient, she was treated with pembrolizumab monotherapy and obtained a durable response. This response is likely entirely attributable to the immunotherapy without any contribution by the previous aborted doxorubicin/olaratumab therapy, as this treatment was only given for one dose several months before the start of immunotherapy.

The expression of different immune-checkpoint receptors varies widely in sarcoma patients depending on the histology. For instance, PD-1/PD-L1 is only expressed in 10% to 20% of cases. The highest expression rate is noticed in non-translocation associated sarcomas, such as DDLPS, UPS or LMS (26, 27).

In SARC 028, expression of PD-L1 by tumor cells was present only in 2 of 40 tumors and both of them responded to treatment, although among responders there were also tumors not expressing PD-L1 (2). Moreover, a report of 2 patients with different types of STS (one with DDLPS and the other with myxofibrosarcoma) who did not express PD-L1 showed responses to immunotherapy with nivolumab and ipilimumab (28). These results suggest that using a single biomarker may not be sufficient to identify tumors destined to respond to ICIs accurately. Multiple biopsies obtained before and during treatment in SARC 028 trial have allowed the identification of immune features of the responders during therapy and shown a correlation of response to treatment with the presence of CD8+ infiltrating lymphocytes (5). Consistent with the critical role of T lymphocytes in curtailing tumor growth, in a preclinical model of osteosarcoma and chondrosarcoma, the depletion of T cells resulted in a markedly accelerated tumor growth and reduced survival, demonstrating that T cells play a role in controlling cancer progression (29, 30). Using immunohistochemistry, Pollack et al. identified UPS and LMS as the most lymphocyte-infiltrated subtypes. Moreover, the authors were able to correlate PD-1/PD-L1 expression with the degree of immune cell infiltration, suggesting that the higher the TILs, the more likely a response to ICIs (31).

TILs infiltration, expression of immune-marker such as PD-L1, TMB and MMRd/MSI status are well-known markers suggesting response to treatment in epithelial cancer or in very immunogenic cancers like melanoma (10–14) (32–34).

Most STSs have a low TMB with a median of 2.5 mutations/Mb, a small subset of STSs (around 5%), however, harbor a high TMB (>20 mutations/Mb). This association is histotype-dependent, with 10-20% of angiosarcomas, LMS and UPS displaying high TMB (8). Rosembaum et al. (35) analyzed 35 angiosarcomas who received ICIs therapy. Among them, 28% were harboring a TMB >10 mutations/Mb. However, genomic and immunohistochemical analyses showed no correlation of TMB or PD-L1 expression, nor the infiltration of lymphocytes with the response to ICIs. Validation in prospective meta-analysis showed that ≥2 predictive biomarkers used together may have more power than a single biomarker (36, 37).

TLS represent one of the most promising biomarkers predicting response to ICIs in different cancer types, including STS (10, 25). In our patient, no TLS were identified, while TILs were abundant and PD-L1 was expressed in the tumor immune microenvironment. Our observations suggest that even in the absence of TLS a robust antitumor immune response can be risen, and are in line with the literature showing that the predictive value of TLS is independent of the presence of CD8+ T cells and other recognized markers (25, 38).

Identification of recurrent mutations by genomic analysis can be added to the existing toolbox of predictive immunotherapy biomarkers (39). One of the mutations present in our patient’s tumor was a loss-of-function mutation in *FAT1*, which encodes a tumor suppressor proto cadherin involved in regulating several key pathways in cancer (40–42), including in sarcomas (43, 44). Interestingly, somatic mutations in *FAT1* have been shown to be positively correlated with high TMB and response to ICI therapy in melanoma and non-small cell lung cancer (45, 46). Although its role as a predictive marker in sarcoma has not been investigated, the durable response of our patient to pembrolizumab and the correlation with other more established predictive markers suggests that *FAT1* could serve as a potential marker to predict response to ICIs in sarcoma patients.

HTS also revealed a loss-of-function mutation in *NOTCH2*. Interestingly, there is evidence in the literature correlating dysregulation of Notch signaling pathway with enhanced immunogenicity and increased infiltration of TILs and CD8+ T cells in different cancer types (47, 48), although its role in the immunogenicity of sarcomas still needs to be investigated.

Moreover, a widespread multi-omics analysis in more than 32 cancer types from The Cancer Genome Atlas (TCGA) dataset identified a genomic signature of 11 genes, correlated with TMB, able to predict the response to immunotherapy, which included *FAT1* and *NOTCH2*, both mutated in our patient’s tumor (49).

## Conclusion

Here we described the case of a long-lasting complete response to pembrolizumab in a patient with an undifferentiated MMRd/MSI cardiac sarcoma.

Although immunotherapy may have a role in some STS patients, the identification of suitable candidates who can obtain a meaningful benefit from treatment requires improvement.

Considering the genomic variability between sarcomas and the heterogeneous response to ICIs treatment, validation of new predictive markers is essential to improve the efficacy of immunotherapy in sarcomas. The use of a combination of existing biomarkers may improve their predictive power. This might allow for a more personalized approach and an increased response rate to immunotherapy in diseases that are still considered resistant.

To the best of our knowledge, complete response to immunotherapy in cardiac sarcoma patients is rare. This case report provides the evidence on how a correct histopathologic and molecular characterization of such tumors can lead to a real benefit in sarcoma patient outcome.

## Data Availability

The data analyzed in this case report contains confidential patient information but can be made available upon request.
